# Retrospective analysis of demographic factors in COVID-19 patients entering the Mount Sinai Health System

**DOI:** 10.1371/journal.pone.0254707

**Published:** 2021-07-16

**Authors:** Abrisham Eskandari, Agnieszka Brojakowska, Malik Bisserier, Jeffrey Bander, Venkata Naga Srikanth Garikipati, Lahouaria Hadri, David Goukassian, Kenneth Fish

**Affiliations:** 1 Cardiovascular Research Center, Icahn School of Medicine at Mount Sinai, New York, NY, United States of America; 2 Department of Emergency Medicine, Ohio State University Wexner Medical Center, Columbus, OH, United States of America; Azienda Ospedaliero Universitaria Careggi, ITALY

## Abstract

With the continued rise of the global incidence of COVID-19 infection and emergent second wave, the need to understand characteristics that impact susceptibility to infection, clinical severity, and outcomes remains vital. The objective of this study was to assess modifying effects of demographic factors on COVID-19 testing status and outcomes in a large, diverse single health system cohort. The Mount Sinai Health System de-identified COVID-19 database contained records of 39,539 patients entering the health system from 02/28/2020 to 06/08/2020 with 7,032 laboratory-confirmed cases. The prevalence of qRT-PCR nasopharyngeal swabs (χ^2^ = 665.7, p<0.0001) and case rates (χ^2^ = 445.3, p<0.0001) are highest in Hispanics and Black or African Americans. The likelihood of admission and/or presentation to an intensive care unit (ICU) versus non-ICU inpatient unit, emergency department, and outpatient services, which reflects the severity of the clinical course, was also modified by race and ethnicity. Females were less likely to be tested [Relative Risk(RR) = 1.121, p<0.0001], and males had a higher case prevalence (RR = 1.224, p<0.001). Compared to other major ethnic groups, Whites experienced a higher prevalence of mortality (p<0.05). Males experienced a higher risk of mortality (RR = 1.180, p = 0.0012) at relatively younger ages (70.58±11.75) compared to females (73.02±11.46) (p = 0.0004). There was an increased severity of disease in older patient populations of both sexes. Although Hispanic and Black or African American race was associated with higher testing prevalence and positive testing rates, the only disparity with respect to mortality was a higher prevalence in Whites.

## Introduction

In late December 2019, the first cases of Coronavirus disease 2019 (COVID-19), caused by the novel severe acute respiratory syndrome coronavirus 2 (SARS-CoV2), were reported in Wuhan, Hubei Province, China [1]. The virus has affected more than 213 countries and territories with significant morbidity and mortality and thus was declared a pandemic by the World Health Organization in March 2020 [1]. As of June 2021, there are 178,360,849 confirmed cases of COVID-19 and 3,869,384 deaths were reported in the United States (US) accounting for 33,190,195 confirmed cases and 596,003 deaths globaly [2]. Similar to SARS-CoV (2002–2003) and Middle East respitory syndrome (MERS)-CoV epidemics, COVID-19 is associated with severe respiratory complications, including viral pneumonia and acute respiratory distress syndrome (ARDS) [3, 4]. Additionally, COVID-19 has been associated with cardiovascular disease (CVD) manifestations, such as myocardial injury, arrhythmias, coagulopathies, and acute coronary syndrome [3, 4]. Exacerbated immunopathogenic responses trigger oxidative damage, chronic inflammation, and, ultimately, multiorgan failure in COVID-19 patients [3, 4]. Despite the rapid research advances, the predisposing factors to infection, severity, and morbidity to COVID-19 remain unclear. Understanding risk modifiers is critical to managing global infection and treatment strategies, especially for those with pre-existing chronic diseases.

Multiple studies from different countries have shown an age-associated vulnerability to CoV2 infection in older individuals. Reports from China, Korea, Italy, the United Kingdom, and New York City have presented evidence for increased risk of mortality with increase in age. This includes higher case fatality rates (CFR) and deaths per 100,000 individuals in aging populations [5]. Based on each country’s self-reporting publicly available data, as of November 19, 2020, Korea’s Agency for Disease Control and Prevention’s study reported 11,314 patients with confirmed cases of COVID-19 with 6.76% CFR in patients 70–79 years of age and 19.46% CFR in those ≥80 years of age. This is in contrast to the overall CFR of 1.67% [6]. A similar study of 44,672 patients from China by their Center of Disease Control and Prevention showed an overall CFR of 2.3% yet a CFR of 8.0% in the 70–79 years old patients and a CFR of 14.8% in the ≥80 years old patients [7]. Similarly, Italy had a CFR of 16.9% in the 70–79 years old population and 24.4% in the ≥80 years old population [8]. Despite Italy’s pattern mimicking that of China and Korea, its overall CFR was significantly higher at 9.2%. However, this could be due to different population characteristics with respect to age. According to PEW Research Center analysis of United Nations data, Italy has a median age of 47 with 30% of its population being ≥60 years of age [9]. In China and South Korea, the median age is 38 and 44 with 17% and 23% of the population being ≥60 years of age, respectively. Data from the United Kingdom and New York City (NYC) have provided similar results with exponential growth in deaths per 100,000 individuals [10, 11].

While the increased risk of mortality with increased age seems unanimously apparent, the modifying effects of age on susceptibility to infection and impact on COVID-19 disease course is not well-understood. Korea’s cases per 100,000 individuals showed peaks in the 20–29 years old age range as well as a smaller second maximum in the ≥80 years old patients [6]. In contrast, New York showed steadily increasing cases per 100,000 individuals, peaking in the ≥75 years old patients [10]. Relative illness ratio (RIR) studies in Korea seem to present the same pattern mimicked by China. Italy and Spain’s RIR trends were similar to that of NYC. This could very likely be due to testing availability and rates as well as unstandardized detection and reporting system(s) in different countries. While China and Korea performed aggressive CoV2 testing despite symptom status, underprepared and overwhelmed systems in the US, Italy, and Spain were initially offering tests to only those considered severely symptomatic. During the first wave of the pandemic, the Centers for Disease Control and Prevention (CDC) advised patients who were asymptomatic or had mild symptoms to self-quarantine in an attempt to avoid exhaustion of resources.

Data comparing sexes with respect to cases and CFR in China has also highlighted disparities between males and females in the context of CoV2 infections [12]. While by absolute number of cases there seems to be a small difference between the sexes, hospitalization in men far exceeds that of women with 50% more hospitalized men, indicating men likely have a more severe clinical course [13–15] and likely require ICU care. Comparison of CFR across genders in Europe shows a higher likelihood of mortality in men. In addition, Spain, Switzerland, Italy, China, and Germany all showed similar male to female CFR ratios that fell in the 1.7–1.8 range [12].

Racial disparities in CoV2 infections have been widely reported. Communities of color, specifically Blacks and Hispanics, experience higher proportions of cases and hospitalizations compared to their representative portion of the general population (11,15). NYC has one of the most diverse populations in the world, that can provide a valuable data for reported differences among races. Based on earlier regularly updated news reports that should be taken with significant caution as they may not stand vigorous scientific testing, in non-hospitalized, non-fatal hospitalized, and confirmed deceased patients, when comparing cases per 100,000 by race/ethnicity, the trend is overwhelming and consistent with Blacks having the highest rates, followed by Hispanics, then Whites, and lastly Asians. The risk of mortality was also elevated compared to their White counterparts during the same period. These trends, while less severe, persisted when adjusting for age and relevant clinical as well as social variables [16]. Additionally, a study of 11,210 individuals with laboratory-confirmed COVID-19 in 92 hospitals across 12 states showed no increased risk in all-cause hospital mortality of Black patients compared to White patients after adjusting for sociodemographic factors and comorbidities [17]. This suggests that demographics may modify the likelihood of patients entering hospital systems for COVID-19.

NYC rapidly became one of the early major epicenters of the US pandemic [2]. The Mount Sinai Health System (MSHS) has played a leading role in response to the COVID-19 crisis in New York and the US. The MSHS de-identified database is one of the largest and most racially diverse populations collected from 5 major hospitals across four boroughs (Manhattan, Bronx, Queens, and Brooklyn) [18]. Therefore, the MSHS database represents an ideal data source to study patients’ risks for hospitalization, a critical step to developing epidemiological models that provide long-term COVID-19 projections and lead to improved infection prevention strategies, countermeasures, and treatment strategies.

In this retrospective study, we analyzed a de-identified MSHS COVID-19 patient database generated from 80,108 patients with a COVID-19 concern, including 109,000 individual service encounters in the MSHS from February 28^th^ to June 8^th^ 2020. Here, we provide detailed epidemiological and statistical analyses to determine the relationship between ethnicity, sex, and age on the clinical severity and mortality in the setting of COVID-19.

## Materials and methods

### Electronic medical record data mining

The MSHS, with hospitals and outpatient sites throughout the metropolitan NYC area, was at the epicenter of the global COVID-19 pandemic. A de-identified MSHS COVID-19 patient database was generated from the electronic medical record (EMR; EPIC) system and made available to the MSHS research community. From February 28th to June 8th, 2020, this database consisted of 80,108 masked electronic record numbers (representing individual patients) that entered the MSHS with a COVID-19 related encounter, including 109,000 individual service encounters, including outpatient (OP) visits which entail telehealth and urgent care visits, emergency department (ED) visits, and inpatient (IP) admissions. IP admissions were further categorized into non-ICU or ICU admission. For the remainder of this manuscript, non-ICU patients will be referred to as IP. Multiple variables were featured in the database and used in stratification and characterization of the cohort’s associated COVID-19 risk. These included age, sex, race, ethnicity, SARS-CoV2 qRT-PCR testing, encounter types, location of care, admission type codes, discharge locations, and deceased indicators. Consistent with the database, only ethnic groups represented in the US Census were included in our analyses: White, Hispanic, Black or African American, Other, Asian, Native Hawaiian or Pacific Islander, and American Indian or Alaskan native. Black or African Americans will be referred to as Black for the remainder of this manuscript. A combined race and ethnicity variable defined by Mount Sinai’s COVID Disparities and Equity Taskforce was used to stratify demographic information to better capture Hispanic patients. Considering this combined variable, we will refer to race and ethnicity simply as ethnicity for the remainder of this manuscript.

### Data preparation: COVID-19 severity score

To reflect CoV2 infection severity, we created a scoring system based on the patient’s clinical service encounter where "unspecified" was scored as 1, OP visits represented mild cases were scored as 2, ED visits scored as 3, IP encounters scored as 4, and finally to ICU encounters, which represented the most severe clinical courses scored as 5. Unspecified service encounters, such as radio-oncology and neuropsychiatric visits (n = 67 patients), were excluded. After a severity score was calculated for each encounter, only the first encounter with the maximum severity score was selected to represent an individual patient’s clinical course. Thus, each patient was represented only once based on an encounter representing the most severe portion of their clinical score to avoid multiple representations in our analyses. We excluded encounters that included unspecified/unidentified data with respect to age, sex, and ethnicity. This filtration, in addition to the removal of patients with unidentified service encounters, limited our analysis to include 39,539 patients.

## Assessing sex and racial disparity in CoV2 testing rates

We analyzed the associations between ethnicity and sex on CoV2 testing. With regards to CoV2 nasopharyngeal qRT-PCR testing status, the database included individuals who tested positive (CoV2+), presumed positive, negative (CoV2-), and individuals who were not tested. Presumed positive indicates that a patient’s positive qRT-PCR result has not yet been confirmed by the Center for Disease Control and Prevention (CDC). Within our cohort of 39,539 patients, 17.2% were CoV2+, which include presumed positives, 49.4% were CoV2-, and 32.8% were not tested for the SARS-CoV2 antigen. We performed a Chi-square (*Χ*^*2*^) test for homogeneity to assess the association between race and testing under the null hypothesis (H_0_). The distribution of tested versus not tested individuals was the same among four major races—White, Hispanic, Black, and Asian. Additionally, we performed a *Χ*^*2*^ test under the H_0_ where the distribution of CoV2+ versus CoV2 individuals is the same among the four ethnicities. We looked for these associations in the cohort and conducted a separate analysis with a focus on the total deceased population (n = 1,912). Native Hawaiian or Pacific Islander and American Indian or Alaskan Native were excluded from this analysis as their sample size was limited, making the *X*^*2*^ distribution potentially not valid. Furthermore, the Other ethnic population was excluded as it consists of a diverse group of ethnicities, which would limit the interpretation of our analysis. With regards to sex, two independent relative risk analyses were done to assess for a significant difference in the likelihood of females not being tested to males not being tested, and females being CoV2+ and males being CoV2+.

### Methods for assessing sex and ethnic disparity in mortality rates

Further statistical analyses were performed to compare severity and mortality as stratified by sex and ethnicity. For the remainder of our analysis, we focused on patients with positive (n = 6,537 patients) or presumed positive (n = 495 patients) qRT-PCR results (i.e. CoV2+ patients). Additionally, we excluded all patients for which an outcome was unknown, limiting the analysis to 6,924 patients—5,629 individuals who recovered and 1,295 deceased. The count of recovered versus deceased individuals is reflective of results as of June 8^th^, 2020. Considering our calculations were performed based on COVID-19 recovered and deceased populations, we further filtered our cohort based on patients with known clinical outcomes i.e., discharged or deceased. Considering 93.4% of our deceased patient population is captured in the IP and ICU units, we removed patients whose discharge location was 1) unknown ("blank", n = 101) or 2) involved "admission to inpatient" (n = 7). The latter category represented ED patients with a single encounter at MSHS that did not end up being admitted to IP for unclear reasons. Of all patients discharged to hospice (n = 45), 18 were indicated as deceased, and 27 were indicated as still alive. Despite the poor prognosis of hospice patients, we included these patients in our analysis to avoid an assumption on the outcome—they represent 0.5% of all survivors.

### Examination of sex and ethnicity on clinical severity

To evaluate the association between ethnicity and clinical severity, an *Χ*^*2*^ test was performed under H_0_. The distribution of CoV2+ individuals in all clinical services is the same among White, Hispanic, Black, and Asian subgroups. The same analysis was used to detect an association between sex and severity with the H_0_ assuming equal distribution of males and females at each severity. The risk of mortality was compared among ethnicities and then sexes via relative risk (RR) analyses. Relative risk analysis for each race was done against all other races independently with 6 comparisons.

### Age distributions by sex and clinical service

We then looked at age as a modifier of outcomes in the overall CoV2+ population and subsequently in each encounter type corresponding to varying severities. Additionally, individuals born in the MSHS were automatically included in the database and removed from our analysis. Patients greater than 90 years of age were grouped under a single 90+ category and were also excluded from our age distribution frequency plots and statistical analysis. For the female population, this consisted of 171 individuals aged 0 and 532 individuals over 90, while for the male population, this included 182 and 252 individuals, respectively. Assessments for a significant difference in means were conducted using a one-way ANOVA test at the 95% CI using Sidak statistical hypothesis testing to correct for multiple comparisons. Comparisons were performed between varying severities for CoV2+ females, CoV2+ deceased females, CoV2+ males, and CoV2+ deceased males. Inter-sex comparisons were then made without correction for multiple comparisons (Fisher’s LSD Test) as each comparison stands alone. The mean age of females was compared to the mean age of males at every severity. This was done for CoV2+ patients and then with a focus on their deceased subgroup.

### Statistical analysis

All statistical analyses were conducted at the 95% confidence interval using GraphPad Prism version 7.00 for Windows. Significance was assigned at p < 0.05.

## Results

### Ethnicity composition

**Fig 1:**
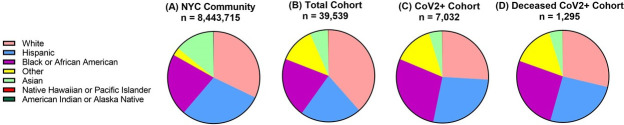
Diversity of Mount Sinai Health System COVID-19 patient population. (A) Ethnic composition of the 2018 New York City catchment population based on US Census Bureau American Community Survey is shown. (B) Mount Sinai Health System cohort is representative of 39,539 patients that entered the health system from February 28^th^ to June 8^th^ 2020 with a COVID-19 encounter, whether diagnosed with COVID-19 or were under investigation for infection with the novel coronavirus. (C) Of the cohort, 7,032 patients had positive or presumed positive CoV2 qRT-PCR, and within this confirmed CoV2 positive population, (D) 1,295 patients were deceased.

We compared the patient population to the overall greater NYC catchment area based on the 2018 community survey US Census data (Table ID: B03002). Of the 39,539 patients, 38.5% of individuals identified as White (n = 15,216), 21.4% as Hispanic (n = 8,465), 21.0% as Black (n = 8,314), 12.5% identified as Other (n = 4,935), 6.4% as Asian (n = 2,536), 0.1% as Native Hawaiian or Pacific Islander (n = 52) and 0.1% as American Indian or Alaskan Native (n = 21) (Fig 1A–1D and Table 1).

**Table 1:** Diversity of Mount Sinai Health System COVID-19 patient population.

Race	NYC Community (%)	Total Cohort (%)	CoV2+ Cohort (%)	Deceased CoV2+ Cohort (%)
**White**	32.1	38.5	25.9	28.7
**Hispanic**	29.1	21.4	27.3	25.6
**Black or African American**	22.0	21.0	28.1	26.1
**Other**	2.8	12.5	13.9	14.7
**Asian**	13.8	6.4	4.6	48.0
**Native Hawaiian or Pacific Islander**	0.03	0.1	0.1	0.1
**American Indian or Alaska Native**	0.2	0.1	0.0	0.0

Race and ethnicity distributions demonstrate the Mount Sinai Health System patient cohort is reflective of the 2018 NYC catchment population. Within the deceased CoV2+ population, there is a comparable mortality observed in the White (28.7%), Hispanic (25.6%), and Black or African American (26.1%) racial and ethnic groups.

Compared to the demographic analysis of the greater NYC area, White and Other patients were overrepresented by 6.3% and 9.7%, respectively, in the MSHS COVID-19 total population (n = 39,539) (Table 1). Furthermore, Hispanic, Black, and Asian populations were underrepresented by 7.7%, 0.9%, and 7.4%, respectively (Table 1). Compared to MSHS COVID-19 cohort (Fig 1B and Table 1), three ethnic groups represented higher percentages of the CoV2+ patients—Hispanics 5.9%, Blacks 7.1%, and Others 1.4% (Fig 1C and Table 1). White and Asian patients represented lower CoV2+ testing percentages, 12.6%, and 1.8%, respectively (Fig 1C and Table 1).

### CoV2 testing by ethnicity and sex

**Fig 2:**
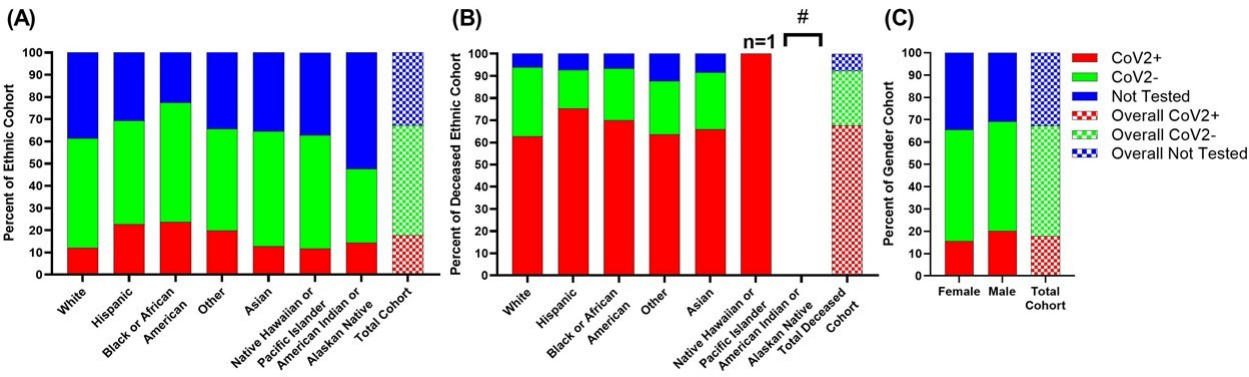
CoV2 testing distribution of Mount Sinai Health System by race and ethnicity and by sex. (A) Of the 39,539 patients captured in the Mount Sinai Health System with a COVID-19 related encounter, the majority of patients across all racial and ethnic groups either tested negative for CoV2 (49.4%) or were not tested (32.8%). Within this population, Black or African Americans (77.5%) and Hispanics (69.3%) had the highest positive test rates. (B) 67.7% of the deceased population tested positive for CoV2, 24.6% tested negative, and 7.6% were not tested. Only 1 individual identified as Native Hawaiian or Pacific Islander is represented in the deceased population and tested positive. In our catchment group, no American Indian or Alaskan Natives passed away (#). Rates of testing were similar across the remaining ethnic and racial groups. (C) CoV2 testing distribution across sexes. The cohort consists of 52.5% females and 47.5% males. Rates of testing were higher in males (69.2%, 12,980/18,769), who also tested positive for CoV2 more than females (p<0.0001).

We also looked at testing rates within sex and race (Fig 2A–2C, and Table 2A–2B). Of the 39,539 patients, 17.8% were CoV2+, 49.4% CoV2-, and 32.8% were not tested (Fig 2 and Table 2A).

**Table 2:** CoV2 testing distribution of Mount Sinai Health System by race and ethnicity across the (A) total patient population and (B) deceased patient population.

**(A)**	**CoV2+**	**CoV2-**	**Not Tested**	**Total Ethnic Cohort**
	**Absolute Number (Percent of Ethnic Cohort)**			
**White**	1,823 (12.0)	7,511 (49.4)	5,882 (38.7)	15,216
**Hispanic**	1,919 (22.7)	3,949 (46.7)	2,597 (30.7)	8,465
**Black or African American**	1,977 (23.8)	4,469 (53.8)	1,869 (22.5)	8,315
**Other**	978 (19.8)	2,263 (45.9)	1,694 (34.3)	4,935
**Asian**	326 (12.9)	1,311 (51.7)	899 (35.5)	2,536
**Native Hawaiian or Pacific Islander**	6 (11.8)	26 (51.0)	19 (37.3)	51
**American Indian or Alaskan Native**	3 (14.3)	7 (33.3)	11 (52.4)	21
**Total Cohort**	7,032 (17.8)	19,536 (49.4)	12,971 (32.8)	39,539
**(B)**	**CoV2+**	**CoV2-**	**Not Tested**	**Total Deceased Ethnic Cohort**
	**Absolute Number (Percent of Deceased Ethnic Cohort)**			
**White**	372 (62.6)	185 (31.1)	37 (6.2)	594
**Hispanic**	332 (75.3)	77 (17.6)	32 (7.3)	441
**Black or African American**	338 (70.0)	113 (23.4)	32 (6.6)	483
**Other**	190 (63.6)	72 (24.1)	37 (12.4)	299
**Asian**	62 (66.0)	24 (25.5)	8 (8.5)	94
**Native Hawaiian or Pacific Islander**	1 (100.00)*	0 (0.00)*	0 (0.00)*	1*
**American Indian or Alaskan Native**	-**	-**	-**	-**
**Total Cohort**	1,295 (67.7)	471 (24.6)	146 (7.6)	1,912

Amongst the total COVID-19 Mount Sinai Health System cohort (n = 39,539 patients), 17.8% tested positive or presumed positive for CoV2, 49.4% tested negative for CoV2, and 32.8% of the population was not tested, possibly due to lack of test availability during the peak of the pandemic. False-negative test rates should be considered based on clinical presentation and supporting clinical evidence.

The individuals who tested negative or were not tested (82.2% of the MSHS cohort) also capture individuals with false-negative qRT-PCR results and individuals with clinical presentation, imaging, and labs consistent with COVID-19. Interestingly, there was a significant association when comparing qRT-PCR testing between the four major ethnic groups. Blacks and Hispanics had higher positive testing rates compared to other groups with 23.8% (n = 1,977) and 22.7% (n = 1,919), respectively (Table 2A).

Rates of positive testing were similar between Whites (12.0%) and Asians (12.9%). Testing prevalence, defined as those who received qRT-PCR out of the entire ethnic population, by ethnicity was as follows—77.6% for Blacks, 69.4% for Hispanics, followed by 64.6% for Asians, and 61.4% for Whites (χ^2^ = 665.7, p<0.0001) (Table 2A). Others, Native Hawaiians or Pacific Islanders, and American Indians or Alaskan Natives were once again excluded from this analysis purely for consistency. In qRT-PCR tested individuals, we found a significant association (χ^2^ = 445.1, p<0.0001) between test results and race. Within the total deceased patient population (n = 1,912) (Table 2B), there is no association between race and testing prevalence (χ^2^ = 0.9088, p = 0.8233), but there was a significant association between race and positive testing rates (χ^2^ = 25.79, p < 0.0001). The testing status in the deceased population is as follows: 67.7% were CoV2+ (n = 1,295), 24.6% Cov2- (n = 471), and 7.6% (n = 146) were not tested (Fig 2B and Table 2B). Within the ethnic groups, CoV2+ rates in the deceased patients were as follows– 62.6% for Whites, 75.3% for Hispanics, 70.0% for Blacks, 63.6% for Others, and 66.0% for Asians (Table 2B). Only 1 patient who identified as Native Hawaiian or Pacific Islander was deceased and tested positive. No American Indian or Alaskan Native passed away. We also looked at the prevalence and rates of positive testing between sexes (Fig 2C and Table 3).

**Table 3:** CoV2 testing distribution across sexes.

	CoV2+	CoV2-	Not Tested		Total Sex Cohort (%)
	Absolute Number (Percent of Sex Cohort)				
**Female**	3,241 (15.6)	10,347 (49.8)		7,182 (34.6)	20,770 (52.5)
**Male**	3,791 (20.2)	9,189 (49.0)		5,789 (30.8)	18,769 (47.5)
**Total Cohort (%)**	**7,032 (17.8)**	**19,536 (49.4)**		**12,971 (32.8)**	**39,539**

Of the total Mount Sinai Health System patient population who presented with a COVID-19 concern, 15.6% of females versus 20.2% of males tested positive for CoV2. Rates of negative testing were similar between both sexes (49.8% for females and 49% for males).

Of the 39,539 COVID-19 related encounters, 20,770 (52.5%) patients were female and 18,769 (47.5%) were male (Fig 2C and Table 3). Within the total populations, 15.6% of females were CoV2+ versus 20.2% of males (Table 3), which could be partially attributed to females being less likely to be tested than males (RR = 0.89, p<0.0001). Indeed, within our study population, more than 34.6% of the females were not tested compared to 30.8% of the males (Table 3). In tested patients, males were more likely to test positive than females (RR = 1.22, p<0.0001). Negative results occurred at similar rates in both sexes (49.8% female and 49% male) (Table 3). These findings may suggest higher susceptibility of males to CoV2 infection.

### Clinical severity by ethnicity and sex

We evaluated the clinical severity of CoV2+ patients (n = 7,032) by ethnic group and sex (Table 4A–4B). COVID-19 disease severity was ranked based on clinical service as described in the methods. In the CoV2+ population, representation in each clinical service class is as follows: 10.6% (743/7,032) OP visits, 27.2% (1,911/7,032) ED visits, 49.0% (3,449/7,032) IP and 13.2% (929/7,032) ICU admission (Table 4A–4B). This suggests that MSHS patient population is representative of the wide range of clinical severity of COVID-19. Of note, based on information cataloged in the database, it is unclear if asymptomatic patients were captured. However, due to initial advisories by the CDC for asymptomatic and mildly symptomatic patients to stay home and self-quarantine, it is likely that a much larger of these patients never presented into the hospital system. Of the CoV2+ patients (total of 7,032), 1,295 individuals passed away, with 93.4% (1,209/1.295) of the mortality observed in hospitalized patients, including IP (20.2%, 698/3,449) and ICU admission (55%, 511/929) (Table 4A–4B).

**Table 4:** COVID-19 mortality in Mount Sinai Health Systems stratified by race and ethnicity and by sex.

**(A)**		**Clinical Service (Deceased CoV2+ / Total CoV2+)**				
		**Outpatient**	**Emergency Department**	**Inpatient**	**ICU**	**Total (%)**
**Race and Ethnicity**	**White**	0 / 318	26 / 419	226 / 871	120 / 215	372 / 1,823 (20.4)
	**Hispanic**	2 / 173	19 / 499	156 / 964	155 / 283	332 / 1,919 (17.3)
	**Black or African American**	0 / 112	22 / 664	180 / 965	136 / 236	338 / 1,977 (17.1)
	**Other**	1 / 102	11 / 247	103 / 477	75 / 152	190 / 978 (19.4)
	**Asian**	0 / 37	5 / 81	32 / 167	25 / 41	62 / 326 (19.0)
	**Native Hawaiian or Pacific Islander**	0 / 1	0 / 0	1 / 4	0 / 1	1 / 6 (16.7)
	**American Indian or Alaskan Native**	0 / 0	0 / 1	0 / 1	0 / 1	0 / 3 (0)
	**Grand Total (%)**	**3 / 743 (0.4)**	**83 / 1,911 (4.3)**	**698 / 3,449 (20.2)**	**511 / 929 (55.0)**	**1,295 / 7,032 (18.4)**
	**(B)**					
		**Clinical Service (Deceased CoV2+ / Total CoV2+)**				
		**Outpatient**	**Emergency Department**	**Inpatient**	**ICU**	**Total (%)**
**Sex**	**Male**	2 / 409	51 / 936	374 / 1,851	323 / 595	750 / 3,791 (19.8)
	**Female**	1 / 334	32 / 975	324 / 1,598	188 / 334	545 / 3,241 (16.8)
	**Grand Total (%)**	**3 / 743 (0.4)**	**83 / 1,911 (4.3)**	**698 / 3,449 (20.2)**	**511 / 929 (55.0)**	**1,295 / 7,032 (18.4)**

(A) Distribution of deceased patients in the Mount Sinai Health System by race and ethnicity and clinical service. The severity of CoV2 infection was evaluated based on clinical service sought by patients, with outpatient visits representing a mild clinical course and ICU admission representing a severe clinical course. Of the 7,032 patients captured in the Mount Sinai Health System with qRT-PCR confirmed or presumed positive CoV2, 1,295 patients passed away, with most deaths captured in the inpatient (n = 698 patients) and ICU (n = 511 patients) admissions. Across all races and ethnicities, most deaths are captured in individuals who sought emergency care or were admitted as inpatients or in the ICU. 67 patients were captured in the “Other” service class, which was reflective of neuropsychology, and radio-oncology visits were excluded from the analysis. (B) There is a higher representation of males in inpatient and ICU admissions; however, mortality across both genders in each unit is similar.

Mortality within each ethnic group was as follows: 20.4% of Whites, 17.3% of Hispanics, 17.1% of Blacks, 19.4% of Others, 19.0% of Asians, 16.7% of Native Hawaiian or Pacific Islander, and no mortality was reported amongst American Indians or Alaskan Natives (Table 4A).

We performed further subgroup analysis of clinical severity within each ethnic group (Table 4A). To this extent, we looked at the total CoV2+ patients in a given clinical unit over the total CoV2+ cases in each respective ethnic group. Within the White population, the highest representation of individuals was seen in the IP (47.8%, 871/1,823), followed by ED (23.0%, 419/1,823), OP (17.4%, 318/1,823), and ICU admission (11.8%, 215/1,823). Of the total CoV2+ population, Whites comprised 42.8% of OP visits. Within the remaining ethnic groups, the trend for representation in each service class was the same in the order of IP, ED, ICU admissions, and OP visits. These ethnic populations vary from the White population in their predominance of ICU patients over OP visits. IP admission within each ethnic group was as follows: 50.2% (964/1,919) for Hispanics, 48.8% (965/1,977) for Blacks, 51.2% (167/326) for Asians, and 48.8% (477/978) for Others. ED visits within each ethnic group were: 23.0% (499/1,919) for Hispanics, 33.6% (664/1,977) for Blacks, 24.9% (81/326) for Asians, and 25.3% (247/978) for Others. ICU admissions within each ethnic group were: 14.7% (283/1,919) for Hispanics, 11.9% (236/1,977) for Blacks, 12.6% (41/236) for Asians, 15.5% (152/978) for Others. OP visits within each ethnic group were: 9.0% (173/1,919) for Hispanics, 5.7% (112/1,977) for Blacks, 11.4% (37/326) for Asians, and 10.4% (102/978) for Others. Overall, between the White, Black, Hispanic, and Asian ethnic groups, there was a significant association between race and severity (χ^2^ = 183.1, p<0.0001) (Table 4A). In addition, we also detected significant association between sex and severity (χ^2^ = 57.59, p<0.0001) (Table 4B). The smallest difference between sexes and severity was identified in the ED patients (49.0% (936/1,911) of males as opposed to 51.0% (975/1,911) of females). While the proportions of OP and IP patient were comparable between sexes (55.0% (409/743) of males vs. 45.0% (334/743) of females were OP, and 53.7% (1851/3449) of males vs. 46.3% (1,598/3,449) of females were IP), the biggest difference between sexes and severity was identified in ICU patients. The ICU’s held 64.0% (595/929) of males versus 36.0% (334/929) of females (Table 4B).

### COVID-19 mortality by ethnicity and sex

**Fig 3:**
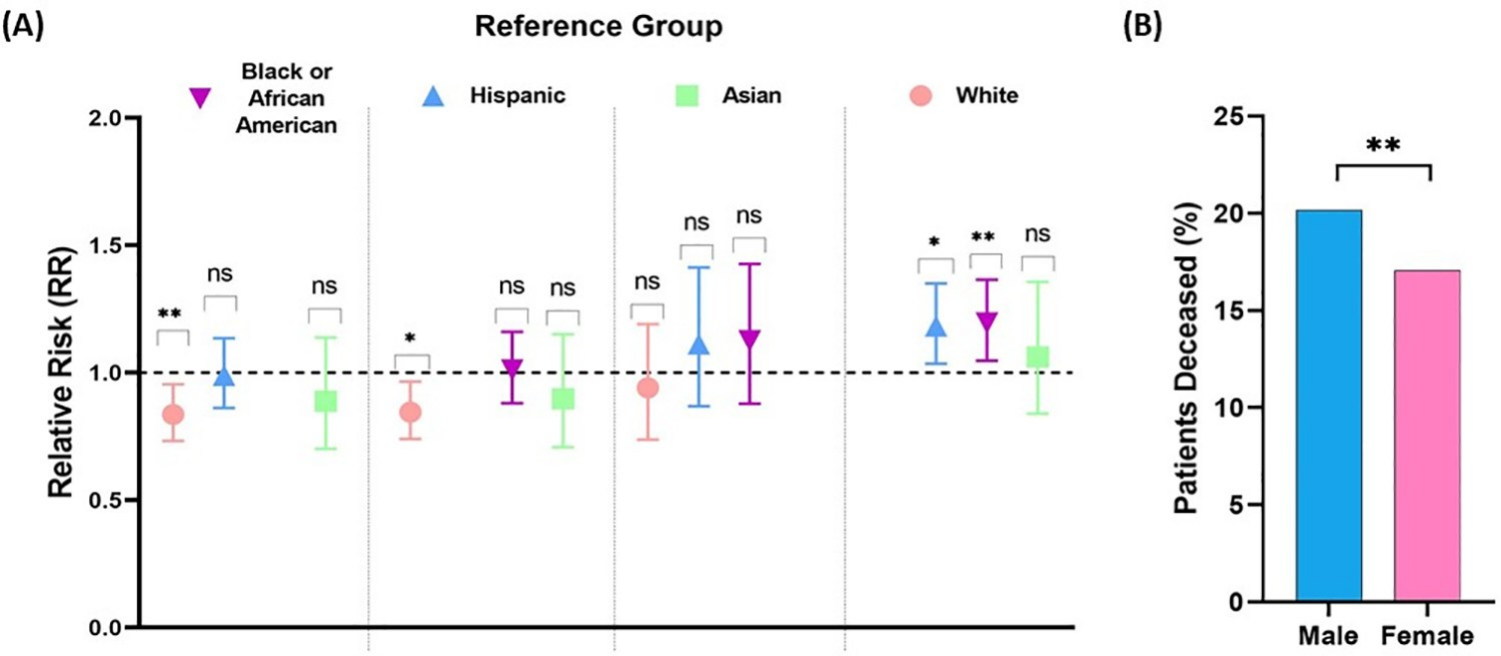
COVID-19 mortality in Mount Sinai Health Systems stratified by race and ethnicity and by sex. (A) Relative risk of mortality from COVID-19 stratified by race and ethnicity. Whites have a higher relative risk of mortality from CoV2 infection relative to the Black or African American and Hispanic population. There was no significant change in the risk of mortality between the White and Asian population. Reciprocally, both the Black or African American and Hispanic populations have a lower relative risk of mortality relative to the White population. The risk of mortality was not significant between Black or African Americans, Hispanic, and Asian populations. Given the limited population size of the representative Native Hawaiian or Pacific Islander and American Indian or Alaskan Native populations, subgroup analysis of the relative risk of mortality could not be assessed. Relative risk (RR) calculations and Fisher’s exact test for significance were performed; **p < 0.01, *p = <0.05, ns = not significant. (B) Relative risk of mortality from COVID-19 stratified by sex. Amongst the deceased population, the risk of mortality was 1.188 -fold greater in the male population (p = 0.0008).

We calculated the relative risk of mortality in CoV2+ patients for each ethnic group to further assess for any racial disparities with regards to mortality (Fig 3A and 3B). We found that only the White population had a significantly increased relative risk of mortality compared to both Blacks (RR = 1.196, p = 0.0086) and Hispanics (RR = 1.183, p = 0.0134) (Fig 3A).

There was no significant change in the risk of mortality for the White population relative to the Asian population. Additionally, the risk of mortality was not significant between Black, Hispanic, and Asian populations (Fig 3A). These findings suggest the White population has a higher risk of mortality in the setting of CoV2 infection compared to other ethnic groups in the MSHS cohort, albeit, our data is not age-adjusted. In addition to their higher CoV2+ testing results indicated above, males had a 1.19-fold greater risk of mortality than females (males: 20.12%, females: 17.05% p = 0.0012) (Fig 3B). Taken together, these findings indicate that in the MSHS cohort, males were infected at higher rates than females and were at greater risk of mortality outcomes.

### Age distribution stratified by sex

**Fig 4:**
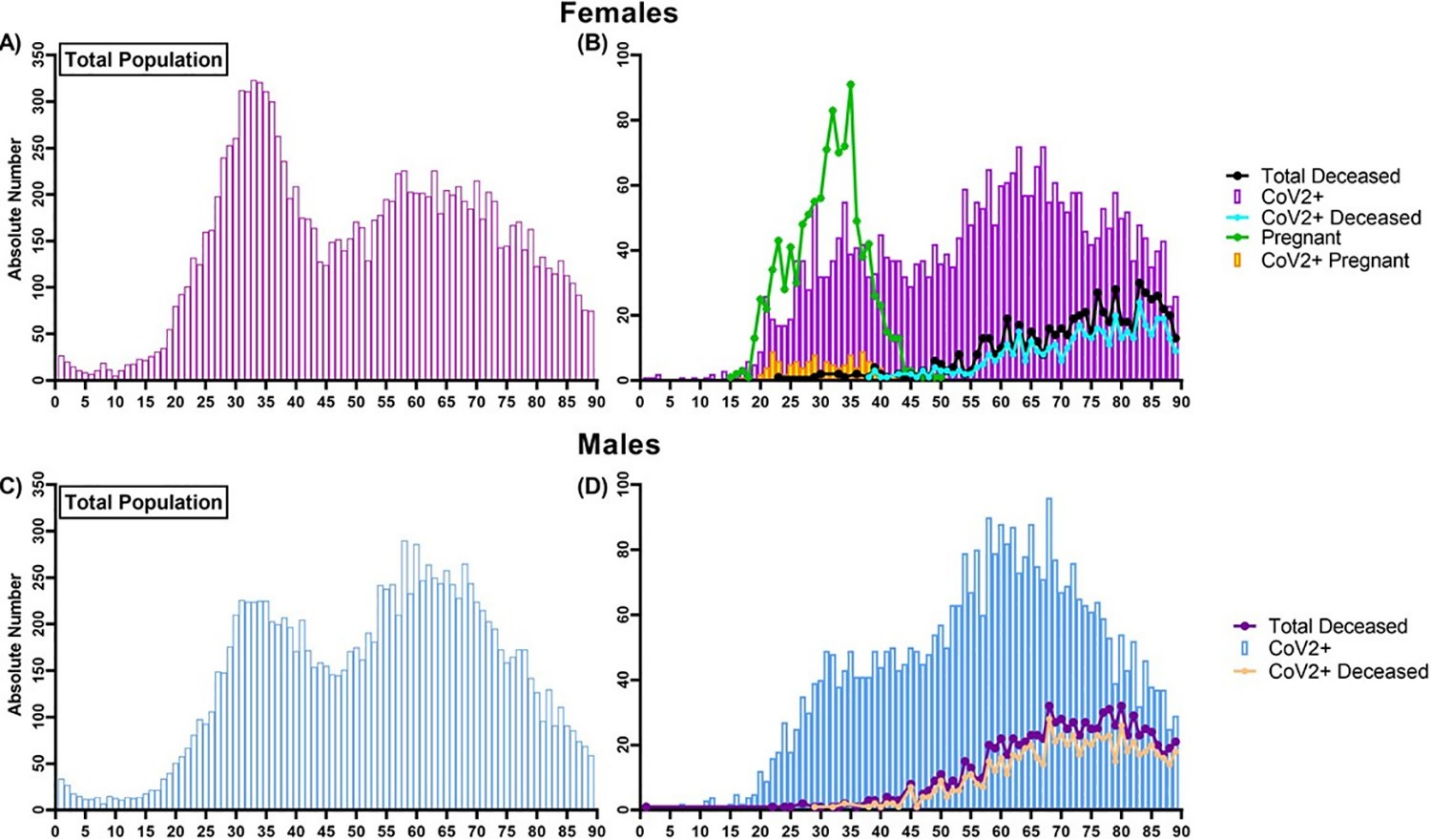
Distribution of Mount Sinai Health System patient cohort by age, sex, mortality, and CoV2 status. (A, C) In the Mount Sinai Health System cohort (n = 39,625) and the qRT-PCR confirmed or presumed positive CoV2 population; there is a bimodal nature of age distribution in both sexes when looking at the affected population. (B) Within the total female population, a large portion of the younger maxima [age = 31 years of age] represents females from labor and delivery (n = 1,068). Within the pregnant population, 10.8% of females tested positive for CoV2+, 85.2% tested negative. Unlike the total population, the age distribution in the deceased COVID-19 positive population is bell-curved skewed right towards the older population in both sexes with an average age of (B) 73±12 years of age for females and (D) 71±12 years of age for males.

We assessed the age distribution of patients that entered the MSHS for a COVID-19 concern by sex. Age distribution in total patients, regardless of testing status, is bimodal. Notably, the age distribution of the total CoV2+ population is, although attenuated at first maxima, still bimodal for both sexes (Fig 4A–4C). For females, the two maxima occur around 33 and 61 years of age, while for males, the maxima occur at approximately 32 and 58 years of age (Fig 4A–4C). The average age for female patients was 58±19, and for males, 57±17 years of age (Table 5). Of note, within the total female population, a large portion of the younger maxima (31 years of age) represents females who presented for labor and delivery (n = 1,068) (Fig 4B, green line). Interestingly, in the total deceased population and deceased CoV2+ population, the age distribution in both sexes resembles a skewed bell curve shifted toward the older patient population with an average age of 73±12 years of age for CoV2+ females and 71±12 years of age for CoV2+ males, suggesting a small (2-year) but statistically significant difference in the average age of death from COVID-19 between males and females (p = 0.0022).

**Table 5:** Age comparison by gender across severity.

	Females		Males	
	Average Age ($\bar{x}$ ± SD)			
	Total CoV2+	Deceased CoV2+	Total CoV2+	Deceased CoV2+
**Outpatient**	46 ± 16	69 ± 0	45 ± 17	69 ± 1
**Emergency Department**	50 ± 17	73 ± 12	49 ± 17	73 ± 11
**Inpatient**	63 ± 19	76 ± 10	63 ± 16	74 ± 11
**ICU**	65 ± 14	69 ± 12	62 ± 14	67 ± 12
**Overall**	**58 ± 19**	**73 ± 12**	**57 ± 18**	**71 ± 12**

The average age of females compared to males in OP, ED, IP, and ICU settings. Average age summaries include results for the total CoV2+ population as well as results for the subsect of the patients that were deceased for both males and females. Overall results for both genders are also included.

**Fig 5:**
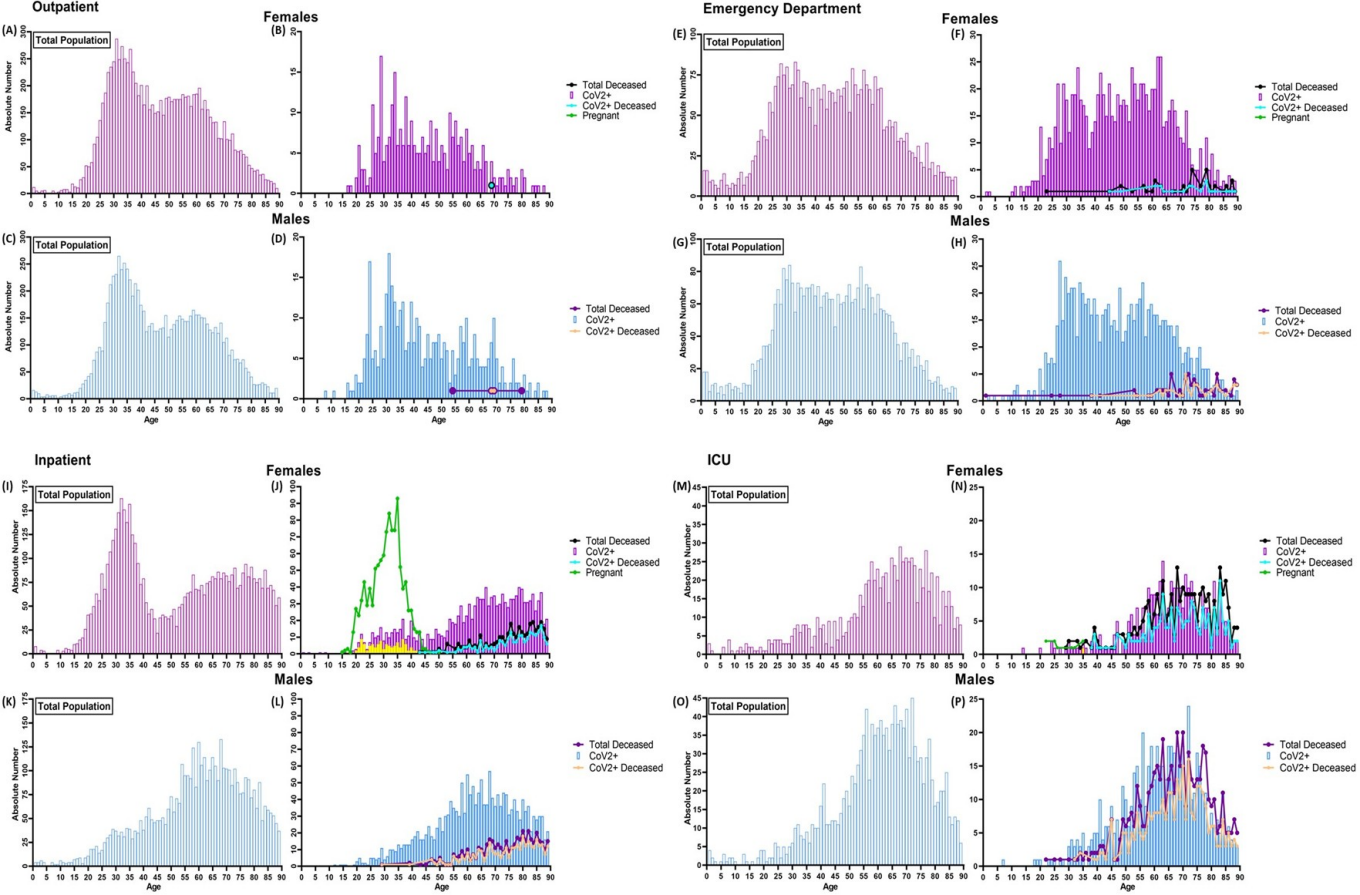
Distribution of Mount Sinai Health System deceased patients by age, sex, and clinical service. As in the MSHS cohort (n = 39,625 patients), there is a bimodal nature of age distribution in both sexes when looking at the combined affected population within (A-D) outpatient and (E-H) emergency department setting. Only 3 individuals passed away in the outpatient setting, and 83 individuals in the emergency department; in both services, mortality was higher in the elderly population. (B) Within the outpatient setting, the average age of positive or presumed positive females is 46±16 years of age with maxima of 31 years of age and 54 years of age, respectively. (D) The average age of positive or presumed positive males is 45±17 years of age, with maxima occurring at 31 and 59 years of age, respectively. (F) Within the emergency department, the average age of positive or presumed positive females is 50 ±17 years of age with maxima of 34 years of age and 62 years of age, respectively. (H) The average age of positive or presumed positive males is 49 ±17 years of age, with maxima occurring at 27 and 59 years of age, respectively. (I, J) The total female inpatient population exhibits a bimodal distribution, where maxima occur around 33 years of age, which consists mostly of females admitted under labor and delivery. The age distribution of deceased inpatient females remains skewed towards the elderly population. (K, L) Unlike the total female population, the age distribution in the inpatient male population is bell-curved, with maxima skewed toward the older population. The age distribution of deceased inpatient males is unchanged. (M-P) Within the ICU, the age distribution of the affected and deceased populations resembles a bell-curved skewed towards to older population with an average age of 69 for females and 67 for males, suggesting older populations exhibited a more severe clinical course. Of note, patients that were 0 years of age (newborns) and 90+ years old were grouped as a method for de-identifying the data and were removed from the analysis.

We assessed age distributions as a function of clinical course severity using our severity scoring system described in the methods as a surrogate for the severity of clinical course (Fig 5A–5P). Across both sexes, the age distribution in the OP (Fig 5A–5D) and the ED (Fig 5E–5H) settings remained bimodal in all patients. However, when focusing on the CoV2+ patients, in OP males and females, age distributions are skewed to the left, with larger number of younger patients testing positive (Fig 5B and 5D).

In the CoV2+ OP population, the average age was 46±16 with maximum peak at 29 years of age for females (Fig 5B), and 45±17 with maximum peak occurring at 31 years of age (Fig 5D). There was no significant difference in the average age between CoV2+ males and females within the OP population (p = 0.9963). The CoV2+ male and female ED patients have less pronounced bimodal age distributions mimicking that of the total male and female ED patients (Fig 5F and 5H). In the ED patients, the average age of CoV2+ females was 50±17, with maxima occurring at 29 and 54 years of age (Fig 5F), whereas the average age of CoV2+ males was 49±17, with maxima occurring at 29 and 56 years of age (Fig 5H). There was no significant difference in the average age between both sexes in the total CoV2+ ED population (p = 0.9274). Within the deceased patients in the ED, there was also no significant difference in average age between sexes (Fig 5F and 5H).

The total IP female population showed a bimodal age distribution, with the early maxima being contributed primarily to females admitted for labor and delivery (Fig 5J), as noted above (n = 1,068). Interestingly, the age distribution for CoV2+ females remained bimodal, with an average age of 63±19 and maxima occurring at 29 and 73 years of age (Fig 5J). Unlike the total CoV2+ population, the age distribution in the CoV2+ female IP population resembles a bell curve skewed right with an average age of 76±10 years of age (Fig 5J). For CoV2+ males, the age distribution in both the total IP and IP deceased population resembles a right-skewed bell-curve with an average age of 63±16 and 74±11 years of age, respectively (Fig 5L). Overall, in the CoV2+ IP population, males appear to be passing away at a significantly younger age than females (p = 0.032). Within the ICU, the age distribution of the total CoV2+ and deceased patients for both sexes is skewed right, suggesting a more severe clinical course for the elderly population (Fig 5, 5J and 5L). In the ICU, there was no significant difference in the average age 65±14 of total females versus males 62±14, or the average age of a CoV2+ deceased female (69 ±12 years of age) versus male (67±12 years of age).

Within the CoV2+ female population, patients admitted as IP or ICU were significantly older compared to females who presented to OP (Fig 5A–5D) or ED (Fig 5E–5H) (p<0.0001), and there was no difference in age between females admitted in IP (Fig 5I–5L) or ICU (Fig 5M–5P) units. Despite this, females in the ICU died at a significantly younger age compared to their respective population admitted as IP (p<0.0001). Similar patterns are observed in the CoV2+ males, with older individuals being hospitalized (IP or ICU admission) compared to the remainder of the population (p<0.0001) (Fig 5K–5L and 5O–5P, Table 5).

Relatively younger aged males (67±12) were dying in the ICU compared to those in the ED (73±11, p = 0.013) and males in the IP population (74±11, p<0.0001) (Table 5). Overall, this suggests that older males exhibit a more severe clinical course. However, higher mortality is skewed towards a relatively younger population.

## Discussion

Since the initial reports of COVID-19 in early December 2019, the novel coronavirus outbreak continues to strain modern society, and its pathogenesis remains to be fully elucidated. Using one of the largest US patient populations across a range of clinical services, including OP, ED, IP, and ICU admission, we assessed the associations between various demographic factors including age, sex, ethnicity on CoV2 infection testing, clinical severity, and mortality. Of 39,539 patients who entered the MSHS with a COVID-19 concern as of June 8^th^ 2020, 7,032 patients (17.9%) were positive or presumed positive for CoV2, of which 1,295 patients passed away (18.4%), and 5,737 recovered and were discharged (81.6%). Lack of testing can possibly be attributed to the shortage of nasopharyngeal swab testing kits during the peak of the pandemic in NYC.

While major ethnic groups are well represented, the differences between census data and the MSHS patient population could be due to the catchment areas for the census data and socioeconomic stratification. Major ethnic groups are well-represented, but more detailed analyses are needed to understand the effects of ethnicity and potential disparities on COVID-19 patients entering the health system.

With regards to CoV2 testing, we found that relative to other ethnic groups, Blacks and Hispanics had higher positive testing and infection rates, which is consistent with prior reports [19]. Although the prevalence of positive testing was higher in these ethnic groups at MSHS, it did not translate to an increased risk of mortality. No association between race and testing prevalence was detected in the 1,912 deceased CoV2+ patients hospitalized at MSHS. Interestingly, a higher risk of mortality was observed in the White patient population compared to other ethnic groups. In our study, elevated risks of mortality in the White patients may be attributed to several factors, included but not limited to—1) an inaccurate representation of COVID-19 cases in MSHS compared to the overall population; 2) due to lack of age adjustment and predominance of the older patient population in the given ethnic group resulting in relative risk calculations which may not be fully representative, ie., within the White population, the average age of deceased hospitalized patients was approximately 75 while in the Black or Hispanic populations this was 72 and 68 respectively; 3) the difference in the average age of hospitalized patients for different ethnic groups, i.e., 75.6% of hospitalized White patients were 70+ years old, while in the remaining ethnic groups individuals age 70+ composed approximately 50% of their hospitalized individuals. An earlier assessment of only inpatients at MSHS by Wang et al. had not observed racial disparities in mortality [19], possibly given the study captured a limited window of patients early in the pandemic with a focus only on the hospitalized population, whereas our data capture several months of patients across varying clinical services. Higher risk of mortality may also be reflective of a higher proportion of hospitalized patients developing sequelae associated with higher morbidity and mortality, including acute respiratory distress (ARDS) [20] and acute myocardial infarction (AMI) [21, 22]. Within the hospitalized White population, 0.9% of patients developed ARDS and 2.0% had AMI, compared to remaining ethnic groups where these sequelae occurred in approximately 0.4% and 1.3% of individuals, respectively. Although we classified clinical severity by service class, the extent of organ involvement or dysfunction is unclear. A limitation of our analysis is that each ethnic group’s predisposition to the development of certain sequelae is unknown highlighting the need for further investigation.

We also found that males tested positive more often than females and had a higher risk of mortality. In comparing case rates, the potential effect of false negatives and unconfirmed cases between both sexes should be taken into consideration. However, the impact of sex on susceptibility to CoV2 infection cannot be entirely excluded in our studies. Our findings regarding increased mortality in CoV2+ males are consistent with prior reports [12], suggesting that sex may modify CoV2 infection or susceptibility to experiencing disease caused by this infectious agent. Indeed, intrinsic biological differences may be contributing to higher susceptibility to infection and poorer outcomes in males compared to females. Among other factors, an innate difference in the activation of a downstream adaptive immune responses in females [23–25], including elevated cytotoxic T cell activation and upregulated expression of antiviral and pro-inflammatory genes such as interferon-gamma (IFNG) that have estrogen response elements [26]. In addition, the sex-associated difference in the modulation of the renin-angiotensin-aldosterone system by estrogen with a particular upregulation of angiotensin-converting enzyme 2 (ACE2) [27, 28] could explain some of the differences between males and females. In addition, estrogen also modulates the ACE2/Ang 1-7/MAS axis, which acts as a counter-regulatory pathway against the classic inflammatory RAAS and may also contribute to the cardioprotective effects of estrogen in females [28]. Thus, female sex hormones may aid in the risk reduction for worse outcomes in the setting of COVID-19. Prospective, well-designed studies are required to elucidate the impact of sex on COVID-19 susceptibility and mortality.

We observed a bimodal age distribution in male and female CoV2+ population with maxima occurring at 33 and 61 years of age for females and 32 and 58 years of age for males. Mortality was highest in the older population for both sexes, with males dying younger (average 71 years of age) than females (average 73 years of age). This bimodal distribution was not observed in IP or ICU populations in both sexes, suggesting that a moderate to severe clinical course is observed predominantly in older patients. Interestingly, the average age of mortality is similar in both sexes for individuals captured in the ICU; however, younger males were passing away in IP compared to females. Overall, these findings suggest that aside from increased susceptibility of infection with CoV2, a more severe disease course in hospitalized males could be attributed to the sex differences noted earlier.

There are several limitations to our study worth highlighting. We did not age adjust in our relative risk analysis, which may be one of the reasons for the greater risk of mortality that we found in White patients. Furthermore, we included hospice patients in our relative risk analysis. Hospice patients not indicated as deceased are included in the recovered category. However, they contain a very small portion of survivors or recovered patients (0.5%). Representing them in such a way only presents a 2.1% reduction in deceased patients. Our analysis also included all ED and OP populations. The limitations with these patients, as well as with IP and ICU patients post-discharge, is that there is a likelihood of being lost to follow-up as well as the risk of discharge proceeding death. While other studies have used hazard functions to circumvent this issue, they have focused on hospitalized populations. We believe our study allows for better assessment of looking at risks in the overall population. Risks may still be overstated as many individuals remain asymptomatic or present mild symptoms and therefore do not appear in hospital systems. Another limitation in our and many other studies is the false-negative rate of qRT-PCR tests, with studies estimating the sensitivity of most tests to be 70% [29].

## Abbreviations

COVID-19: Coronavirus disease 2019
SARS-CoV2: Severe acute respiratory syndrome coronavirus 2
ACE2: angiotensin-converting enzyme 2
ARDS: Acute respiratory distress syndrome
CVD: cardiovascular disease
CFR: case fatality rates
ED: emergency department
ICU: intensive care unit
IFNG: interferon-gamma
IP: inpatient
RIR: Relative illness ratio
OP: Outpatient
